# More than just noise: careless responding and its systematic effects on reliability, validity, and measurement invariance

**DOI:** 10.3389/fpsyg.2026.1815225

**Published:** 2026-05-15

**Authors:** Levent Ertuna, Gülden Kaya-Uyanik, Duygu Gençaslan

**Affiliations:** Department of Educational Measurement and Evaluation, Faculty of Education, Sakarya University, Sakarya, Türkiye

**Keywords:** careless responding, composite sensitivity index, data quality, instructed response items, insufficient effort responding, measurement invariance, psychometric properties, reverse-coded items

## Abstract

**Introduction:**

Careless responding, in which survey participants fail to attend to item content, is a well-recognized threat to the quality of self-report data. Although prevalence estimates commonly fall between 8 and 12% in student samples, the extent to which careless responding distorts the psychometric properties of attitude measures has received limited attention, particularly outside Western samples.

**Methods:**

The present study investigated the prevalence and psychometric consequences of careless responding in a paper-and-pencil sample of 1,112 Turkish university students who completed the sustainable development awareness scale. An instructed response item embedded within the scale identified 126 respondents (11.33%) as careless, and two post-hoc indicators (longstring and even-odd consistency) converged with this classification. Parallel analyses on the unscreened and screened samples were conducted to evaluate effects on internal consistency, confirmatory factor analysis, multigroup measurement invariance, criterion-related validity correlations, and an item-level Composite Sensitivity Index (CSI).

**Results:**

Internal consistency was higher in the screened sample, with the gains concentrated in the two subscales containing reverse-coded items. Confirmatory factor analysis indices trended in the direction of better fit after screening, and criterion-validity correlations with related constructs were modestly larger in the screened sample at both the manifest and latent levels. Multigroup measurement invariance testing across attentive and careless responders supported metric but not scalar invariance, pointing to systematic intercept differences consistent with acquiescent responding among careless respondents. The composite sensitivity index combining changes in item means, item-total correlations, and factor loadings was used to rank items by vulnerability to careless responding, with results robust to an alternative standardized aggregation. All six reverse-coded items appeared among the ten most sensitive items, despite constituting only one-sixth of the scale, and the item immediately following the attention check showed a notably elevated sensitivity pattern that we interpret as a tentative hypothesis worth further investigation.

**Discussion:**

These findings underscore the importance of routine screening for careless responding and offer practical guidance on the placement of attention checks and the use of reverse-coded items.

## Introduction

Self-report surveys remain the primary means of data collection across the social and behavioral sciences, enabling researchers to gather information on psychological constructs such as attitudes, beliefs, and awareness with relatively low cost and effort (Haeffel and Howard, 2010; Schwarz, 1999). The growing adoption of online survey platforms has broadened the reach of these tools while also reducing the logistical burdens of data entry and administration (Ward and Meade, 2023). Yet whether surveys are administered online or through traditional paper-and-pencil methods, a well-documented concern persists: not all respondents engage with survey items thoughtfully, and those who do not can compromise the quality of the data on which research conclusions rest (Huang et al., 2012; Meade and Craig, 2012; Ward and Meade, 2023).

This concern is captured by the term careless responding, which refers to instances in which survey participants fail to read or adequately process item content, producing responses that do not reflect their actual standing on the constructs of interest (Meade and Craig, 2012; Ward and Meade, 2023). The phenomenon has been described under various labels, including random responding (Beach, 1989), insufficient effort responding (Huang et al., 2012), protocol invalidity (Johnson, 2005), and content nonresponsivity (Nichols et al., 1989). Although these labels differ in emphasis, they converge on a common idea: careless responding constitutes a form of response invalidity that is distinct from other biases such as social desirability or impression management (Maniaci and Rogge, 2014; Meade and Craig, 2012). Whereas social desirability involves deliberate selection of favorable responses, careless responding stems from a lack of motivation to engage with the task at all, contributing error variance that can obscure genuine construct-related information. Moreover, careless responding is not necessarily a stable individual trait; evidence suggests that it can fluctuate within individuals across time and across different portions of a survey (Camus, 2015), further complicating detection efforts.

Prevalence estimates for careless responding range widely, from as low as 3% to as high as 46% depending on sample characteristics and the detection criteria used (Berry et al., 1992; Johnson, 2005; Meade and Craig, 2012; Oppenheimer et al., 2009). In typical research samples, the modal rate has been estimated at 8% to 12% (Curran, 2016; Huang et al., 2012; Meade and Craig, 2012). Rates tend to be higher in settings where respondents have little personal investment in the task, and conditions such as unproctored online administration, lengthy questionnaires, and mandatory participation requirements have all been linked to greater careless responding (Buchanan, 2000; Johnson, 2005; Meade and Craig, 2012). Even at seemingly modest prevalence rates, however, simulation studies have shown that including 5% to 10% careless responders in a dataset can meaningfully alter the conclusions of statistical analyses (Credé, 2010; Woods, 2006).

The cognitive basis for understanding why respondents lapse into careless behavior is provided by Krosnick's (1991) satisficing framework. Optimal responding to a survey item requires the respondent to read the item, retrieve relevant self-knowledge, form a judgment, and translate that judgment onto the response scale (Tourangeau et al., 2000). Each of these stages demands cognitive effort and motivation. Respondents who are unable or unwilling to invest this effort may satisfice, selecting a response that seems acceptable without fully processing the item. At its extreme, satisficing becomes careless responding, in which the respondent abandons thoughtful engagement altogether. Ward and Meade (2023) emphasized that careless responding is best understood as a state influenced by survey, respondent, and environmental characteristics rather than a fixed trait, although Bowling et al. (2016) provided evidence that individual differences in conscientiousness, agreeableness, and emotional stability predict careless responding with some temporal stability.

Several survey-level and environmental factors have been identified as contributors to careless responding. Longer surveys place greater demands on respondent attention, and research has documented declining response times and increasing response invariability as participants progress through lengthy instruments (Berry et al., 1992; Galesic and Bosnjak, 2009; Meade and Craig, 2012). At the item level, reverse-coded items appear particularly susceptible to careless responding because they require an additional cognitive step to process the negation, a step that careless respondents are unlikely to perform (van Sonderen et al., 2013; Woods, 2006). Environmental conditions such as the absence of proctoring, distractions during test administration, and perceived anonymity have also been associated with reduced data quality (Johnson, 2005; Meade and Craig, 2012).

Researchers have developed a range of strategies for detecting careless responders, and these can be grouped into two broad categories: *a priori* methods requiring advance planning and *post hoc* methods that are applied to the completed data (Curran, 2016; Meade and Craig, 2012). *A priori* methods include instructed response items that direct participants to choose a particular option, bogus items presenting implausible statements, self-report attention items, and response time monitoring (DeSimone et al., 2015; Huang et al., 2012; Meade and Craig, 2012). *Post hoc* methods include consistency indices comparing responses to semantically related items, longstring analysis counting runs of identical responses, intra-individual response variability (Dunn et al., 2018), and multivariate outlier detection such as Mahalanobis distance (Costa and McCrae, 2008; Johnson, 2005; Meade and Craig, 2012; Meijer and Sijtsma, 2001). Because different methods tend to flag overlapping but non-identical sets of respondents, multiple indicators are generally recommended for multi-method screening (Curran, 2016; DeSimone et al., 2015). More recently, machine learning approaches have been explored for detecting complex patterns of inattention, though their generalizability across diverse survey contexts remains an open question (Ozaki, 2024; Schroeders et al., 2022).

The psychometric consequences of careless responding have been documented at multiple levels of analysis. At the item level, careless responses introduce error variance that weakens inter-item correlations and depresses reliability estimates (Huang et al., 2012; Maniaci and Rogge, 2014). At the construct level, the picture is more nuanced. Reverse-coded items occupy a particularly complex role in this context: while they help control acquiescence bias, their higher reading difficulty makes them especially sensitive to careless responding. Their presence also allows researchers to distinguish valid from invalid straightlining (Reuning and Plutzer, 2020). Woods (2006) demonstrated through simulations that when even 10% of respondents answered reverse-coded items carelessly, confirmatory factor analyses rejected valid unidimensional models and produced spurious method factors. Kam and Meyer (2015) extended this line of work by showing that careless responding, combined with acquiescence bias, can alter the apparent dimensionality of psychological scales, leading researchers to misinterpret artifacts of response behavior as substantive factors. More recently, Kam (2019) and Arias et al. (2023) have shown with real-world data that careless respondents distort factor loading patterns and inflate inter-factor correlations, threatening both convergent and discriminant validity.

The effects of careless responding are not limited to structural analyses. Because careless responses lack construct-related information, they attenuate observed correlations between measures, which in turn elevates the risk of Type II errors (Credé, 2010; Maniaci and Rogge, 2014). Maniaci and Rogge (2014) reported that screening out inattentive participants could increase statistical power by 5 to 7 percentage points, demonstrating that the gain in precision can more than compensate for the loss in sample size. Under certain conditions, however, careless responding may introduce bias in the opposite direction. When careless responders gravitate toward a common response option such as the scale midpoint, their shared response pattern can create artificial associations between unrelated measures, inflating Type I error rates (Huang et al., 2015). Taken together, these findings indicate that careless responding can both mask real effects and produce spurious ones, making screening necessary regardless of the direction of the anticipated effect.

Despite these well-established threats, routine screening for careless responding remains uncommon across many areas of psychological research (Huang et al., 2015). This gap is notable given that a large share of studies in the field rely on self-report questionnaires administered to student samples under conditions that are known to promote inattentive responding. The literature on careless responding has been built primarily on personality inventories and measures drawn from organizational and social psychology (Curran, 2016; Huang et al., 2012; Meade and Craig, 2012), leaving questions about whether the same patterns hold for other types of scales, including those assessing attitudes and awareness. Similarly, the vast majority of this research has been conducted with North American and Western European samples. Cultural differences in response styles and attitudes toward survey participation mean that prevalence rates and psychometric effects documented in Western contexts may not generalize straightforwardly to other settings (Kay and Saucier, 2023; Ozaki, 2024). In student samples from Turkish universities, for instance, Erdem-Kara and Akbulut (2025) found that different detection methods identified varying proportions of careless responders, with reverse item consistency checks flagging as many as 20% of participants while self-report items proved largely ineffective. Their study also showed that removing careless respondents improved model fit indices more consistently than it improved reliability coefficients, underscoring the particular importance of screening for studies that employ confirmatory factor analysis or structural equation modeling.

Despite this accumulating evidence, the literature on careless responding has focused primarily on individual psychometric properties in isolation, and integrated demonstrations examining effects across reliability, factorial validity, criterion validity, and measurement invariance within a single study remain relatively scarce (Stosic et al., 2024). In addition, the most of this research has been conducted with North American and Western European samples (Huang et al., 2012; Meade and Craig, 2012), leaving open the question of whether the same patterns emerge in cultural contexts where response styles and attitudes toward survey participation may differ (Grau et al., 2019; Kay and Saucier, 2023). Recent methodological advances have expanded the detection toolkit, including model-based approaches that incorporate response time information (Ulitzsch et al., 2022), supervised machine learning techniques (Pokropek, 2026; Schroeders et al., 2022), and comprehensive reviews of attention check methods (Muszyński, 2023), but these newer approaches have not yet been widely applied to examine downstream psychometric consequences.

The present study was designed to address these gaps through a comprehensive investigation of how careless responding affects the psychometric properties of a widely used attitude measure. The sustainable development awareness scale (SDAS; Atmaca et al., 2019), a 36-item instrument measuring awareness of sustainable development across economic, social, and environmental dimensions, provides a suitable testing ground for several reasons. The scale contains six reverse-coded items that are expected to be differentially sensitive to careless responding, it uses a multifactorial structure amenable to confirmatory factor analysis and measurement invariance testing, and it represents a construct domain that has not been previously examined in the careless responding literature.

The study contributes to the existing body of work in four ways. First, it moves beyond the narrow focus on reliability and factor structure that characterizes most prior investigations by examining a broad set of outcomes, including measurement invariance across attentive and careless response groups, criterion-related validity, and the sensitivity of group comparisons to screening decisions. Second, it introduces the composite sensitivity index (CSI), a heuristic that integrates changes in item means, item-total correlations, and factor loadings to rank items according to their overall vulnerability to careless responding. Third, it extends the literature to a non-Western context by examining careless responding among Turkish university students, contributing to the growing body of cross-cultural evidence on data quality issues. Fourth, it provides practical guidance for survey designers by documenting how item characteristics, particularly reverse coding and proximity to attention checks, are associated with differential sensitivity to careless responding.

The specific aims of the study were: (1) to estimate the prevalence of careless responding and to examine whether demographic characteristics predict it; (2) to quantify the effects of careless responding on internal consistency, factorial validity, composite reliability, and average variance extracted; (3) to test measurement invariance across response quality groups; (4) to assess the impact on criterion-related validity correlations and regression analyses; (5) to identify and characterize the items most sensitive to careless responding using the CSI; and (6) to evaluate whether screening alters the detection of meaningful group differences.

On the basis of prior theory and evidence, we expected that roughly 10% of the sample would be identified as careless responders. We hypothesized that screening would improve reliability estimates and yield better confirmatory factor analysis fit. We further expected that criterion-related validity correlations would strengthen after screening, because careless responses on the SDAS were expected to take predominantly random or midpoint forms that introduce construct-irrelevant variance and dilute genuine associations with external criteria (Credé, 2010; Maniaci and Rogge, 2014). Although careless responding can in principle either inflate or attenuate observed correlations depending on the specific mechanism involved (Stosic et al., 2024), attenuation is the more likely direction when the primary instrument contains reverse-coded items (which pull careless responders' scale means toward the midpoint) and the criterion measures use different response formats and contain no reverse-coded items. We anticipated that reverse-coded items would exhibit the greatest sensitivity to careless responding, as reflected in higher CSI values. Finally, we expected that measurement invariance testing would reveal intercept-level differences between attentive and careless responders, consistent with the view that careless responding introduces systematic bias rather than purely random noise.

## Method

### Participants

A total of 1,119 undergraduate students were recruited from nine programs within the Faculty of Education at a large public university in northwestern Türkiye during the 2024–2025 academic year. Seven cases (0.63%) were removed as univariate outliers based on standardized SDAS total scores exceeding |*z*| > 4 (Stevens, 2009), yielding an unscreened sample of *N* = 1,112. Outlier detection preceded careless responding screening for two reasons. First, the two phenomena are conceptually distinct: univariate outliers typically reflect unusually extreme but internally consistent response patterns, whereas careless responding reflects disengagement from item content. Second, inspection of these seven cases revealed that all of them had correctly answered the instructed response item, indicating that they would not have been flagged as careless responders had they been retained. Their inclusion would therefore not have altered the careless responding rate meaningfully (would have been 11.26% rather than 11.33%). This empirical check supports the decision to treat outlier removal and careless responding screening as separate procedures addressing distinct threats to data quality.

The sample comprised 879 women (79.0%) and 233 men (21.0%), with a mean age of 21.3 years (SD = 1.82). Participants were distributed across four academic years: first year (*n* = 217, 19.5%), second year (*n* = 353, 31.7%), third year (*n* = 252, 22.7%), and fourth year (*n* = 290, 26.1%). The nine programs represented were Guidance and Psychological Counseling (GPC; *n* = 232, 20.9%), English language teaching (ELT; *n* = 152, 13.7%), special education (SpE; *n* = 145, 13.0%), preschool education (PE; *n* = 133, 12.0%), classroom teaching (CT; *n* = 115, 10.3%), elementary mathematics teaching (EMT; *n* = 107, 9.6%), science education (SciED; *n* = 96, 8.6%), social studies education (SSE; *n* = 85, 7.6%), and Turkish language teaching (TLT; *n* = 47, 4.2%). Regarding extracurricular engagement, 176 participants (15.8%) reported volunteering for a non-governmental organization, and 542 (48.7%) were members of a university student community.

The sample size was evaluated through sensitivity analyses that account for the actual group size. Given the observed group sizes of *n* = 986 (attentive) and *n* = 126 (careless), a two-tailed independent-samples *t*-test with α = 0.05 and power = 0.80 could detect effects as small as *d* = 0.27. This estimate was computed in *R* with the pwr package (Champely et al., 2020; R package version 1.3-0, R Foundation for Statistical Computing, Vienna, Austria) and G^*^Power 3.1 (Heinrich-Heine-Universität Düsseldorf, Düsseldorf, Germany) (Faul et al., 2007), which yielded an identical result. Comparisons between the unscreened (*N* = 1,112) and screened (*n* = 986) samples, by contrast, involve nested rather than independent samples and are therefore not evaluated using the independent-samples power framework; these comparisons rely on the permutation procedure described in the Section Analytic Strategy. For multigroup confirmatory factor analysis, simulation research indicates that samples of 100 to 200 per group are needed for stable parameter estimation (Wolf et al., 2013); both subsamples met this threshold, although the smaller size of the careless group calls for some caution in interpreting invariance results. The study received approval from the Sakarya University Ethics Committee (Protocol No: E.472092, May 9, 2025), and all participants gave informed consent.

### Measures

#### Sustainable development awareness scale (SDAS)

The SDAS (Atmaca et al., 2019) served as the primary instrument. The scale consists of 36 items across three subscales: economy (13 items), society (nine items), and environment (14 items). Responses are given on a 5-point Likert-type scale ranging from 1 (strongly disagree) to 5 (strongly agree). Six items are reverse-coded (items 1, 8, 10, 24, 31, and 35). The original validation study reported a three-factor structure with acceptable model fit (χ^2^/df = 1.677, RMSEA = 0.040, SRMR = 0.044, GFI = 0.889, IFI = 0.931, TLI = 0.923) and high internal consistency (α = 0.91). Item 26, designed by the scale developers as an instructed response item, directs respondents to select the “Neutral” option and was used as the basis for careless responding detection.

#### Personal social responsibility scale (PSRS)

The PSRS (Davis et al., 2021; Turkish adaptation: Kocar and Seki, 2023) measures perceptions of personal social responsibility across five dimensions: philanthropic, environmental, ethical, legal, and economic responsibility. The scale includes 19 items rated on an 11-point scale from 0 (strongly disagree) to 10 (strongly agree) and contains no reverse-coded items. The Turkish version showed satisfactory psychometric properties (χ^2^/df = 1.91, RMSEA = 0.06, SRMR = 0.06, GFI = 0.90, CFI = 0.94, TLI = 0.93; α = 0.88).

#### Obligation to volunteer as commitment scale (OVC)

The OVC (Gallant et al., 2017; Turkish adaptation: Güzel Gürbüz et al., 2022) is a unidimensional 9-item measure of perceived moral obligation toward volunteering. Items are rated on a 7-point scale from 1 (strongly disagree) to 7 (strongly agree). The Turkish version demonstrated adequate psychometric properties (χ^2^/df = 3.3, RMSEA = 0.09, GFI = 0.95, CFI = 0.97, TLI = 0.93; α = 0.89). Neither the PSRS nor the OVC includes reverse-coded items, which allows us to examine whether careless responding effects extend beyond reverse-keyed content to affect overall response quality across a survey battery.

### Procedure

The study was not preregistered. The analytic plan, variable definitions, and instrument selections were established prior to data collection, but hypotheses and analyses were not formally registered in a public repository. Data collection took place during regular class sessions using paper-and-pencil surveys. Participants were informed about the study through course announcements and took part voluntarily without compensation. The survey booklet began with demographic questions, followed by the SDAS, PSRS, and OVC in fixed order. A research assistant was present throughout each session to address procedural questions. Average completion time was approximately 15 min.

An instructed response item was embedded at position 26 of the 37-item SDAS, placing it at roughly the midpoint of the scale. This location was selected to capture careless responding that may develop as respondents progress through the instrument (Berry et al., 1992; Meade and Craig, 2012). The item explicitly directed participants to select the “Neutral” response option, providing a clear and unambiguous criterion: any response other than “Neutral” indicates a failure to attend to item content (Meade and Craig, 2012; Oppenheimer et al., 2009). Given the total survey length of 65 items (36 SDAS + 1 instructed response + 19 PSRS + 9 OVC), a single instructed response item was considered sufficient, consistent with the recommendation of spacing such items at intervals of roughly 50 to 100 items (Meade and Craig, 2012).

### Additional careless responding indices

To complement the instructed response item classification and to examine convergence across detection methods, two *post-hoc* indices were computed. The longstring index counts the maximum number of consecutive identical responses within a participant's response vector (Johnson, 2005; Meade and Craig, 2012). It was calculated across all 65 survey items using the original (non-reverse-coded) item values, consistent with the rationale that longstring analysis should capture patterns of identical responding as they appear in the respondent's actual answers rather than after analytic recoding. The even-odd consistency index assesses the within-person agreement between odd-numbered and even-numbered items within a scale (Johnson, 2005). It was computed on the 36 SDAS items (excluding the instructed response item) using the careless package in R (Yentes and Wilhelm, 2021), with higher values indicating greater inconsistency and thus a higher likelihood of careless responding. Both indices were used descriptively to examine whether participants flagged by the instructed response item also showed elevated scores on these alternative indicators.

To directly examine whether basing the classification on a single instructed response item understated the prevalence of careless responding and distorted the reported psychometric effects, a supplementary analysis with an extended careless definition was conducted. Under this extended definition, a respondent was flagged as careless if they (a) failed the instructed response item, (b) had a longstring value of 20.72 or higher, or (c) had an even-odd inconsistency score of 1.707 or higher. The two *post-hoc* thresholds were set at two standard deviations above the respective sample means in the unscreened sample. Under this combined criterion, 155 respondents (13.9%) were classified as careless, with 29 additional cases beyond the 126 flagged by the instructed response item alone. The overlap structure among the three indicators is reported in Supplementary Table S5. Reliability, confirmatory factor analysis, measurement invariance, criterion-related correlations, and regression analyses were then repeated under the extended definition, and the results were compared with those obtained from the instructed response item alone. The full results of this supplementary analysis are reported in Supplementary Table S5, and their implications are summarized in the Section Discussion.

### Careless responding detection

Of the 1,112 participants in the unscreened sample, 126 (11.33%) selected a response other than “Neutral” on Item 26 and were classified as careless responders. This rate falls within the 8% to 12% range commonly reported in student samples (Curran, 2016; Meade and Craig, 2012). The screened (attentive) sample thus consisted of 986 respondents (88.67%). Among the 126 careless responders, the majority selected response options in the agreement direction: 63 (50.0%) chose “Agree” and 59 (46.8%) chose “Strongly Agree,” while only 3 (2.4%) selected “Strongly Disagree” and 1 (0.8%) selected “Disagree.” This pronounced skew toward acquiescent responding is consistent with the interpretation that most failures on the instructed response item reflect a tendency to agree with item content without reading it rather than random selection of a response option. Because the instructed response item captures one specific form of careless responding, namely noncompliance with an explicit instruction, the 126 respondents identified in this way should be understood as a subgroup of potentially inattentive participants rather than as an estimate of the total careless responding rate in the sample. Respondents who engage in other forms of inattentive responding, such as consistent midpoint selection or random patterns that nonetheless happen to fall on the instructed category, would not be captured by this single indicator. The practical implications of this boundary are examined through a supplementary analysis that extends the careless definition to include two additional indicators; this analysis is described in the next subsection and its results are reported in Supplementary Table S5.

All psychometric analyses were run in parallel on both the unscreened (*N* = 1,112) and screened (*n* = 986) samples rather than on the screened sample alone. This comparative design allows for direct quantification of the impact of careless responding on every psychometric property examined, and it provides empirical evidence for evaluating whether screening improves data quality and the conclusions drawn from it (Maniaci and Rogge, 2014).

### Analytic strategy

Analyses were conducted in R version 4.5.0 (R Foundation for Statistical Computing, Vienna, Austria) (R Core Team, 2025). Internal consistency was estimated with the psych package (Revelle, 2024). Confirmatory factor analysis was carried out with lavaan (Rosseel, 2012), and measurement invariance testing with semTools (Jorgensen et al., 2022). Effect sizes were computed using effsize (Torchiano, 2020), correlation comparisons with cocor (Diedenhofen and Musch, 2015), and *post-hoc* tests with rstatix (Kassambara, 2023).

Across the analyses reported below, two distinct comparisons are made. The first compares the unscreened sample (*N* = 1,112) with the screened sample (*n* = 986) to quantify how the exclusion of careless responders changes the psychometric properties of the SDAS. Because these two samples are nested, with the screened sample a subset of the unscreened sample, standard independent-samples tests are not appropriate for comparing their means. Instead, comparisons between the two samples use a permutation procedure (Ernst, 2004; Good, 2005; Phipson and Smyth, 2010), which is well suited to situations in which the group-membership label itself is the quantity whose effect is under evaluation. In 10,000 iterations (seed = 2025), the careless-responder label was randomly reassigned to 126 respondents drawn without replacement from the full sample, the full-sample mean and the resulting subsample mean were computed, and their difference was recorded. The observed difference was then compared with the empirical distribution of permuted differences to obtain a two-sided *p*-value, defined as the proportion of permuted absolute differences at least as large as the observed absolute difference. The second comparison treats attentive (*n* = 986) and careless (*n* = 126) respondents as independent groups to examine whether the two response-quality populations differ in their measurement characteristics. Differences in reliability, fit indices, and correlations are summarized using effect-size differences (e.g., Δα, ΔCFI, Δ*r*) alongside conventional significance tests, in recognition of the practical relevance of the observed magnitudes.

#### Preliminary analyses

Chi-square tests of independence assessed whether demographic characteristics (gender, grade level, department, volunteer status, community membership) differed between attentive and careless responders. Cramér's *V* quantified association strength. Binary logistic regression was used to identify demographic predictors of careless responding, with odds ratios and 95% confidence intervals for each predictor. Whereas the chi-square tests examined univariate associations between individual demographic variables and careless responding status, the logistic regression model was estimated to evaluate these associations simultaneously while adjusting for the influence of the other demographic predictors. This multivariable approach allowed us to determine whether any demographic variable remained a significant predictor of careless responding once the shared variance among predictors was accounted for.

#### Reliability

Internal consistency was estimated using Cronbach's alpha (α) with 95% confidence intervals (Feldt, 1965) and McDonald's omega (total – ω*t* and hierarchical – ω*h*). Although omega is generally preferred when essentially tau-equivalent assumption underlying alpha is not met (Dunn et al., 2014), both coefficients are reported here for continuity with broader psychometric literature and with the original SDAS validation study, which reported alpha. Reporting both allows readers to evaluate whether conclusions about reliability depend on the coefficient chosen. ω*h* represents the proportion of total score variance attributable to a single general factor; values above 0.50 support the use of a total score (Reise et al., 2013).

#### Confirmatory factor analysis

The hypothesized structure of the SDAS consists of three correlated latent factors: economy (13 items: SDAS_01, SDAS_02,..., SDAS_13, with SDAS_01, SDAS_08, and SDAS_10 reverse-coded), society (nine items: SDAS_14 through SDAS_22), and environment (14 items: SDAS_23 through SDAS_37 excluding the instructed response item SDAS_26, with SDAS_24, SDAS_31, and SDAS_35 reverse-coded). Each item was specified to load only on its designated factor, and the three factors were allowed to correlate freely. The model was identified by fixing the first indicator loading of each factor to 1.0, and all 36 SDAS items served as observed indicators. This three-factor model was evaluated using robust maximum likelihood estimation (MLR). Fit was assessed with the comparative fit index (CFI ≥ 0.90), Tucker-Lewis index (TLI ≥ 0.90), root mean square error of approximation (RMSEA ≤ 0.08, with 90% confidence intervals), standardized root mean square residual (SRMR ≤ 0.08), and the parsimony-adjusted indices PNFI and PGFI (Hu and Bentler, 1999). Composite reliability (CR > 0.70) and average variance extracted (AVE > 0.50) were calculated following Hair et al. (2019).

#### Measurement invariance

Multigroup CFA was used to test invariance across response quality groups (attentive vs. careless) at three levels: configural (same factor pattern), metric (equal factor loadings), and scalar (equal loadings and intercepts). The ΔCFI ≥ −0.010 and ΔRMSEA ≤ 0.015 criteria recommended by Cheung and Rensvold (2002) and Chen (2007) were used to evaluate each step. Failure to establish scalar invariance would indicate systematic intercept differences between groups, providing evidence that careless responding introduces bias rather than only random error.

#### Group comparisons

Independent samples *t*-tests compared SDAS total and subscale scores between the two samples, with Cohen's *d* and 95% confidence intervals as effect size measures (|*d*| < 0.20 negligible, 0.20 to 0.50 small, 0.50 to 0.80 medium, >0.80 large; Cohen, 1988). Within each sample, *t*-tests were used for dichotomous grouping variables (gender, volunteer status, community membership) and one-way ANOVAs with Games–Howell *post-hoc* tests for polytomous variables (grade level, department). Changes in effect sizes (Δ*d*, Δη^2^) and the number of statistically significant *post-hoc* contrasts were compared across samples. Non-parametric alternatives (Mann–Whitney *U*, Kruskal–Wallis) served as robustness checks.

#### Correlational analyses

Bivariate Pearson correlations among SDAS, PSRS, and OVC total scores were computed for both samples. Fisher's *z*-test compared the magnitude of correlations across samples, and Cohen's *q* quantified the practical significance of the differences (*q* < 0.10 negligible, 0.10 to 0.30 small, 0.30 to 0.50 medium, >0.50 large). Multiple regression models (PSRS subscales predicting SDAS total) were estimated in both samples, with changes in *R*^2^ documented.

#### Item-level sensitivity analysis

To obtain an integrated picture of how careless responding affects individual items, we computed a composite sensitivity index (CSI) for each of the 36 SDAS items. Prior studies have examined changes in item means (Huang et al., 2012), item-total correlations (Credé, 2010), and factor loadings (Kam and Meyer, 2015) as separate indicators of distortion. The CSI brings these three indicators together in a single summary by summing the absolute change in each component between the unscreened and screened samples:


$$\begin{array}{lll} CSI & = & \left|\Delta M|+|\Delta r|+|\Delta \lambda \right| \end{array}$$ (1)

where |Δ*M*| is the absolute difference in item mean, |Δ*r*| is the absolute difference in corrected item-total correlation, and |Δλ| is the absolute difference in standardized factor loading. For Likert-type data scored on a 5-point scale, the three components naturally fall within a comparable numerical range. The |Δ*r*| and |Δλ| components are by definition bounded between 0 and 1, while |Δ*M*| is constrained by the response scale range. In the present data, observed |Δ*M*| values did not exceed 0.15, indicating that the three components operated on roughly comparable scales and supporting the interpretability of their direct summation for item-ranking purposes. The CSI is intended as a practical heuristic for flagging items that may require revision or particular attention rather than as a formal psychometric index with established distributional properties.

To assess the robustness of CSI-based item rankings, a standardized version (CSI-*z*) was also computed. Each of the three components (|Δ*M*|, |Δ*r*|, |Δλ|) was converted to a *z*-score across items before summation, ensuring that all three components contributed equally to the composite regardless of differences in their observed distributions. The agreement between CSI and CSI-*z* rankings was evaluated using Spearman's rank-order correlation and Kendall's tau-*b*. Both indices were reported because they capture slightly different aspects of rank agreement: Spearman's ρ is more sensitive to the magnitude of rank differences, whereas Kendall's τ has a more direct probabilistic interpretation in terms of concordant vs. discordant pairs. Reporting both provides a fuller picture of the robustness of the CSI rankings. High concordance between the two versions would indicate that the rankings produced by CSI are not artifacts of the metric on which the components are measured and that the unstandardized CSI can be used as a practical heuristic without substantive loss of information.

## Results

### Prevalence and demographic predictors of careless responding

Of the 1,112 participants in the unscreened sample, 126 (11.33%) selected a response other than “Neutral” on the instructed response item and were classified as careless responders. The screened sample comprised the remaining 986 attentive respondents (88.67%).

Chi-square tests examined whether careless responding was associated with demographic variables (Table 1). Two significant associations emerged: academic program (Cramér's *V* = 0.14) and student community membership (Cramér's *V* = 0.06). Careless responders were overrepresented in Special Education and Preschool Education, and underrepresented in Science Education. They were also less likely to be members of university student communities. Gender, grade level, and volunteer status were not significantly associated with careless responding.

**Table 1:** Comparison of demographic characteristics between attentive and careless responders.

Variable	Attentive (*n* = 986)	Careless (*n* = 126)	X^2^	df	*p*	Cramér's *V*
Gender						
Female	79.6%	74.6%	1.57	1	0.210	0.038
Male	20.4%	25.4%				
Grade level			2.59	3	0.459	0.048
Department			22.06	8	**0.005**	0.141
Volunteer status			0.16	1	0.686	0.012
Community membership	49.9%	39.7%	4.27	1	**0.039**	0.062

Percentages represent within-group column proportions for attentive (*n* = 986) and careless (*n* = 126) responders. Significant associations (*p* < 0.05) are shown in bold.

Binary logistic regression further explored these patterns (Supplementary Table S1). The full model was significant [$\chi_{\left(14\right)}^{2}$ = 35.58, *p* = 0.001] though the proportion of explained variance was modest (McFadden's pseudo-*R*^2^ = 0.045). With science education as the reference category, several programs showed elevated odds of careless responding: special education [OR = 8.14, 95% CI (2.73, 35.12), *p* < 0.001], Preschool Education [OR = 6.62, 95% CI (2.13, 29.20), *p* = 0.003], English language teaching [OR = 4.31, 95% CI (1.40, 18.87), *p* = 0.023], and social studies education [OR = 3.76, 95% CI (1.07, 17.44), *p* = 0.054]. Community membership was also a significant predictor, with non-members showing higher odds of careless responding [OR = 1.64, 95% CI (1.10, 2.45), *p* = 0.016]. Gender, grade level, and volunteer status did not significantly predict group membership (ps > 0.05).

### Convergence with additional careless responding indices

To examine whether the instructed response item classification converged with alternative *post-hoc* indicators, the longstring index and the even-odd consistency index were computed across the full set of self-report items (see Sections Method, Careless responding detection). The longstring index was substantially higher among respondents flagged as careless (*M* = 15.15, SD = 9.38) than among attentive respondents (*M* = 9.27, SD = 4.21), Welch *t*_(131.5)_ = 6.95, *p* < 0.001, Cohen's *d* = 1.16, 95% CI [0.97, 1.35]. The even-odd consistency score, for which higher values indicate greater within-person inconsistency, was also elevated in the careless group (*M* = 1.10, SD = 1.01) relative to the attentive group (*M* = 0.55, SD = 0.42), Welch *t*_(130.5)_ = 6.01, *p* < 0.001, *d* = 1.06, 95% CI [0.87, 1.25]. The convergence across three conceptually distinct detection methods, the instructed response item, the longstring index, and the even-odd consistency index, supports the validity of the instructed-item classification used as the primary criterion in the present study.

### Reliability

Internal consistency estimates are presented in Table 2. Cronbach's alpha for the SDAS total scale rose from 0.891 in the unscreened sample to 0.918 in the screened sample (Δα = 0.027). At the subscale level, larger differences were observed for economy (α: 0.697 vs. 0.770; Δα = 0.073) and environment (α: 0.784 vs. 0.843; Δα = 0.059), whereas the society subscale, which contains no reverse-coded items, showed essentially no change (Δα = −0.005). Although the Δα value for the total scale is small in absolute terms and unlikely to be of practical consequence on its own, the asymmetric pattern across subscales is informative: reliability differences were concentrated in the two subscales that contain reverse-coded items, suggesting that the influence of careless responding on reliability operates primarily through these items rather than across the scale uniformly. Comparisons of mean SDAS scores between the unscreened and screened samples are reported in the Section Group Comparisons, where the nested structure of the two samples is addressed via a permutation procedure.

**Table 2:** Internal consistency estimates for SDAS scores by sample.

Scale	*k*	α Uns	95% CI (Uns)	α Scr	95% CI (Scr)	Δα	*ωt* Uns	*ωt* Scr	*ωh* Uns	*ωh* Scr
SDAS total	36	0.891	[0.882,0.900]	0.918	[0.911,0.925]	0.027	0.934	0.945	0.781	0.809
Economy	13	0.697	[0.670,0.722]	0.770	[0.748,0.791]	0.073	0.779	0.821	—	—
Society	9	0.843	[0.829,0.857]	0.838	[0.823,0.853]	−0.005	0.877	0.876	—	—
Environment	14	0.784	[0.765,0.802]	0.843	[0.828,0.857]	0.059	0.857	0.888	—	—

K, number of items; Uns, unscreened (N = 1,112); Scr, screened (n = 986); α, Cronbach's alpha; 95% CI, 95% confidence interval for alpha (Feldt, 1965); Δα, α(Scr) – α(Uns); ωt, McDonald's omega total; ωh, hierarchical omega. Hierarchical omega was computed only for the SDAS total scale because the bifactor decomposition required for ωh is meaningful at the level of a multidimensional scale rather than at the unidimensional subscale level. ωh/ωt = 0.86 in the screened sample. Mean differences between samples were tested with a permutation procedure (see Sections Results, Group Comparisons) because the screened sample is nested within the unscreened sample.

McDonald's omega total for the full scale increased from 0.934 to 0.945 following screening, and hierarchical omega rose from 0.781 to 0.809. The ω*h*/ω*t* ratio of 0.86 for the screened sample indicates that a large share of the reliable variance is captured by a general factor, supporting the use of total scores for the SDAS (Reise et al., 2013).

### Confirmatory factor analysis

The three-factor model (economy, society, environment) was fitted separately to both samples using robust maximum likelihood estimation (Table 3). The fit indices were broadly similar across samples but trended in the direction of better fit for the screened sample: CFI was 0.897 vs. 0.910 (Δ = 0.013), TLI 0.891 vs. 0.904 (Δ = 0.013), and SRMR 0.043 vs. 0.041 (Δ = −0.002). RMSEA was essentially unchanged (0.046 vs. 0.047). The ΔCFI of 0.013 exceeds the 0.010 threshold commonly applied in measurement invariance testing (Chen, 2007), suggesting a modest but detectable gain in model-data correspondence. We do not report comparative AIC and BIC values across the two samples because these information criteria depend on sample size and are not directly comparable when the samples differ substantially in *N*.

**Table 3:** Confirmatory factor analysis fit indices by sample.

Sample	χ^2^	df	CFI	TLI	RMSEA	90% CI	SRMR
Unscreened (*N* = 1,112)	1,635.37	591	0.897	0.891	0.046	[0.044,0.049]	0.043
Screened (*n* = 986)	1,464.31	591	0.910	0.904	0.047	[0.044,0.050]	0.041
Δ (Scr – Uns)	—	—	0.013	0.013	0.001	—	−0.002

Three-factor model (economy, society, environment) estimated with robust maximum likelihood. 90% CI, 90% confidence interval for RMSEA. All χ^2^ values significant at p <0.001. AIC and BIC are not reported because they depend on sample size and are not directly comparable when the samples differ in N. Δ represents the difference in fit index values between the screened and unscreened samples and is shown for fit indices that are not sample-size dependent.

**Figure 1:**
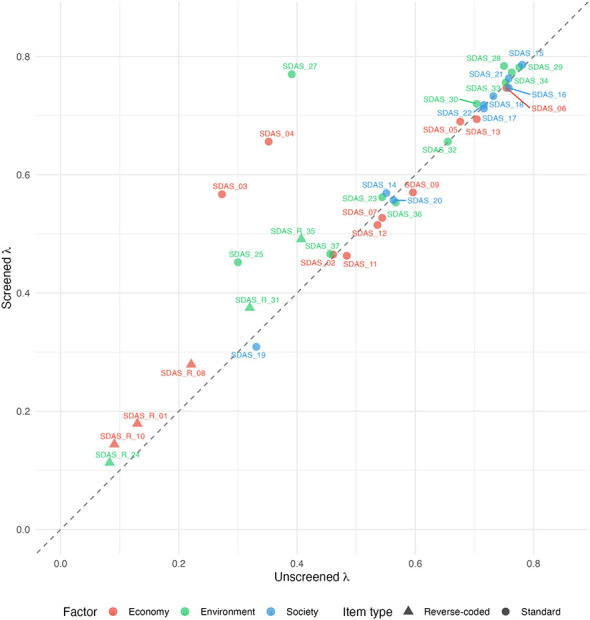
Standardized factor loadings for SDAS items across the unscreened and screened samples. Each point represents a single item, with unscreened sample loadings on the horizontal axis and screened sample loadings on the vertical axis. Points are colored by SDAS factor (economy, society, environment), and reverse-coded items are shown as triangles while standard items are shown as circles. Items above the diagonal line had higher loadings in the screened sample.

Standardized factor loadings (Figure 1; Supplementary Table S2) increased on average from 0.533 in the unscreened sample to 0.573 in the screened sample. Of the 36 items, 25 (69.4%) showed higher loadings after screening. The largest gains were observed for SDAS_27 (Δλ = 0.379), SDAS_04 (Δλ = 0.304), and SDAS_03 (Δλ = 0.294). All six reverse-coded items showed improved loadings following screening, with increases ranging from 0.030 to 0.084.

Composite reliability (CR) exceeded the 0.70 threshold recommended by Hair et al. (2019) for all factors in both samples, with values ranging from 0.775 to 0.887. Average variance extracted (AVE) fell below the conventional 0.50 criterion for the economy and environment factors in both samples. These are the two factors that contain all six reverse-coded items, whose low factor loadings (0.083 to 0.279 in the unscreened sample) pull down the average extracted variance for the factor as a whole. Nonetheless, AVE values improved after screening (economy: 0.244 to 0.284; environment: 0.327 to 0.384), reflecting reduced error at the factor level. The society subscale, by contrast, showed virtually no change in AVE between samples, consistent with its lack of reverse-coded items and its similarly stable Cronbach's alpha.

### Measurement invariance

Multigroup CFA was conducted to test measurement invariance across attentive and careless response groups (Table 4). The configural model served as the baseline (CFI = 0.878, RMSEA = 0.055). Although the CFI fell below 0.90, the configural model provided an adequate reference point for evaluating relative changes in fit across invariance steps, which is the primary purpose of the sequential testing procedure (Chen, 2007). Constraining factor loadings to equality (metric model) produced minimal change (ΔCFI = −0.001, ΔRMSEA = −0.001), satisfying Chen's (2007) criteria for metric invariance. The scalar model, which additionally constrained item intercepts, was not supported (ΔCFI = −0.020, ΔRMSEA = +0.004). A chi-square difference test confirmed significant deterioration from the metric to the scalar step, Δ$\chi_{\left(33\right)}^{2}$ = 362.36, *p* < 0.001.

**Table 4:** Measurement invariance tests across response quality groups.

Model	χ^2^	df	CFI	RMSEA	ΔCFI	ΔRMSEA	Supported
Configural	2,765.83	1,182	0.878	0.055	—	—	Baseline
Metric	2,814.41	1,215	0.877	0.054	−0.001	−0.001	Yes
Scalar	3,118.96	1,248	0.857	0.058	−0.020	0.004	No

Multigroup CFA comparing attentive (*n* = 986) and careless (*n* = 126) responders. Chen (2007) criteria for invariance support: ΔCFI ≥ −0.010, ΔRMSEA ≤ 0.015. Metric invariance supported; scalar invariance not supported, indicating systematic intercept differences between groups. χ^2^ values shown are scaled (Satorra-Bentler) statistics from robust maximum likelihood estimation. The chi-square difference test between the metric and scalar models, Δχ^2^ (33) = 362.36, *p* < 0.001, was computed using the Satorra and Bentler (2001) scaled difference procedure and is not equivalent to the simple difference of the χ^2^ values in the table.

These results indicate that attentive and careless responders interpret the items in a comparable way (equivalent factor loadings) but differ systematically in their level of item endorsement (nonequivalent intercepts). Such intercept differences are consistent with the presence of acquiescence bias among careless responders. Indeed, 96.8% of those who failed the instructed response item selected a response in the agreement direction, indicating a strong tendency to endorse item content without processing it. This pattern would shift intercepts upward for positively worded items and downward for reverse-coded items, producing exactly the kind of scalar non-invariance observed here (Huang et al., 2012; Woods, 2006).

### Group comparisons

*Screened vs. unscreened sample means*. Because the screened sample is a subset of the unscreened sample, the two are nested rather than independent, which rules out the use of standard independent-samples tests for comparing their means. A permutation test with 10,000 iterations (seed = 2025) was therefore used to assess whether mean SDAS scores differed between samples beyond what would be expected by random reassignment of the careless-responder label. Observed mean differences (unscreened minus screened) were small across all scores: SDAS total (Δ = −0.247, *p* = 0.151), economy (Δ = −0.107, *p* = 0.100), and society (Δ = 0.080, *p* = 0.112) showed no reliable differences. For Environment, the observed difference (Δ = −0.220) fell outside the permutation distribution (*p* = 0.007), indicating a small but statistically reliable shift. The direction of this shift is consistent with the interpretation that inattentive responses on reverse-coded environment items pulled the unscreened mean toward the midpoint. Overall, screening changed score distributions only minimally, an important consideration for applied settings where normative comparisons are used.

For gender, women scored higher than men on the SDAS in both the unscreened sample (*d* = 0.289, 95% CI [0.14, 0.44], *p* < 0.001) and the screened sample [*d* = 0.275, 95% CI (0.12, 0.43), *p* = 0.001]. The effect size was stable across samples (Δ*d* = −0.014). Effect sizes for all demographic comparisons are summarized in Supplementary Table S3. Participants who reported volunteering for a non-governmental organization had higher SDAS scores than non-volunteers in both the unscreened sample [*d* = 0.205, 95% CI (0.04, 0.37), *p* = 0.006] and the screened sample [*d* = 0.242, 95% CI (0.07, 0.42), *p* = 0.002], with a slight increase in effect size after screening (Δ*d* = 0.037). Community members also scored higher than non-members in the unscreened sample [*d* = 0.128, 95% CI (0.01, 0.25), p = 0.033] and in the screened sample [*d* = 0.185, 95% CI (0.06, 0.31), *p* = 0.004]. The effect size was larger in the screened sample (Δ*d* = 0.057), although both comparisons were statistically significant at the conventional α = 0.05 level.

One-way ANOVAs revealed a significant grade-level effect in both the unscreened sample, *F*_(3, 1, 108)_ = 4.19, *p* = 0.006, η^2^ = 0.011, ω^2^ = 0.009, and the screened sample, *F*_(3, 982)_ = 5.35, *p* = 0.001, η^2^ = 0.016, ω^2^ = 0.013. Games–Howell *post-hoc* tests identified two significant pairwise differences in the unscreened sample (first vs. second year, *p* = 0.009; first vs. third year, *p* = 0.020). In the screened sample, four pairwise differences were significant: the two that were significant in the unscreened sample plus the contrasts between second and fourth year (*p* = 0.021) and between third and fourth year (*p* = 0.032). The number of significant pairwise contrasts was thus two in the unscreened sample and four in the screened sample.

Department-level ANOVAs yielded a significant effect in both the unscreened sample, *F*_(8, 1, 103)_ = 2.99, *p* = 0.003, η^2^ = 0.021, and the screened sample, *F*_(8, 977)_ = 2.50, *p* = 0.011, η^2^ = 0.020. The slight decrease in effect size (Δη^2^ = −0.001) and the unchanged number of significant *post-hoc* pairs (two in each sample) suggest that the distribution of careless responders across programs did not markedly influence these comparisons.

### Correlational analyses

Manifest correlations between SDAS and the criterion measures are reported in Table 5. SDAS-PSRS was *r* = 0.351 in the unscreened sample and *r* = 0.376 in the screened sample (Δ*r* = 0.025), and SDAS-OVC was *r* = 0.288 vs. *r* = 0.321 (Δ*r* = 0.033). The differences were not statistically significant (Fisher's *z* = −0.65, *p* = 0.519 for SDAS-PSRS; *z* = −0.82, *p* = 0.414 for SDAS-OVC), and Cohen's *q* values were negligible (0.028 and 0.036). The correlation between PSRS and OVC was virtually unchanged (0.541 vs. 0.534).

**Table 5:** Correlations between SDAS and criterion measures by sample.

Relationship	*r* Uns	*r* Scr	Δ*r*	*z*	*p*
SDAS total—PSRS	0.351	0.376	0.025	−0.65	0.519
SDAS total—OVC	0.288	0.321	0.033	−0.82	0.414

Uns, unscreened sample (*N* = 1,112); Scr, screened sample (*n* = 986); PSRS, personal social responsibility scale; OVC, obligation to volunteer as commitment scale. Fisher's z-test used to compare dependent correlations. All correlations significant at *p* < 0.001.

To complement these manifest correlations and to provide measurement-error-corrected estimates, latent correlations were also extracted from a five-factor structural model (Economy, Society, Environment, PSRS, OVC) fitted separately to both samples. As expected, latent correlations were uniformly larger than their manifest counterparts. For example, the latent correlation between Environment and PSRS was 0.444 in the unscreened sample and 0.454 in the screened sample, compared with manifest values of 0.347 and 0.380, respectively. The pattern of small post-screening increases observed at the manifest level held at the latent level as well, with Δ values of 0.005 to 0.025 across the seven SDAS-criterion latent correlations. Detailed latent correlation results appear in Supplementary Table S4.

Multiple regression models predicting SDAS total scores from the five PSRS subscales were estimated for both samples (Table 6). Adjusted *R*^2^ was 0.178 in the unscreened sample and 0.184 in the screened sample (Δ*R*^2^ = 0.006). The pattern of significant predictors was consistent across samples. Environmental Responsibility was the strongest positive predictor (β = 0.243 in the unscreened sample, 0.248 in the screened sample), followed by legal responsibility (β = 0.207 vs. 0.186) and economic responsibility (β = 0.119 vs. 0.117). Philanthropic Responsibility showed a significant negative coefficient in both samples (β = −0.107 vs. −0.084), consistent with a suppressor effect once the remaining dimensions were controlled. Ethical responsibility was not a significant predictor in either sample. The standardized coefficients were similar across samples, with absolute differences in β values of 0.028 or less.

**Table 6:** Multiple regression coefficients predicting SDAS total scores from PSRS subscales.

Predictor	*B* (Uns)	SE (Uns)	β (Uns)	*p* (Uns)	*B* (Scr)	SE (Scr)	β (Scr)	*p* (Scr)
Philanthropic resp.	−0.185	0.057	−0.107	**0.001**	−0.147	0.061	−0.084	**0.016**
Environmental resp.	0.654	0.105	0.243	**<0.001**	0.674	0.111	0.248	**<0.001**
Ethical resp.	0.005	0.070	0.003	0.940	0.060	0.074	0.031	0.422
Legal resp.	0.584	0.097	0.207	**<0.001**	0.523	0.101	0.186	**<0.001**
Economic resp.	0.207	0.058	0.119	**<0.001**	0.204	0.061	0.117	**<0.001**

Resp., responsibility; Uns, unscreened (*N* = 1,112); Scr, screened (*n* = 986); B, unstandardized coefficient; SE, standard error; β, standardized coefficient. The dependent variable in both models is SDAS total score. R^2^ = 0.182 (Uns) and 0.188 (Scr); adjusted R^2^ = 0.178 (Uns) and 0.184 (Scr). Significant coefficients (*p* < 0.05) are shown in bold.

### Robustness of findings to careless responding definition

A supplementary robustness analysis compared the results obtained under the instructed response item classification with those obtained under an extended criterion that combined the instructed response item with the longstring and even-odd consistency indicators (see Section Method and Supplementary Table S5). Direction of change relative to the unscreened sample was consistent across the two definitions for all four Cronbach's alpha coefficients, all four confirmatory factor analysis fit indices, and the SDAS-OVC correlation. The measurement invariance pattern was identical under both definitions: metric invariance was supported (ΔCFI = −0.002 under the instructed-item classification and −0.004 under the extended criterion) and scalar invariance was not (ΔCFI = −0.020 and −0.021, respectively). Two outcomes did not change in the same direction across the two definitions. The SDAS-PSRS correlation increased modestly after instructed-item screening (Δ*r* = +0.025) but showed essentially no change under the extended criterion (Δ*r* = −0.003). Similarly, the variance explained by PSRS subscales in the regression predicting SDAS total increased slightly after instructed-item screening (Δ*R*^2^ = +0.007) but decreased under the extended criterion (Δ*R*^2^ = −0.020). Complete results are presented in Supplementary Table S5, and the methodological implications of this pattern are discussed below.

### Item-level sensitivity analysis

The composite sensitivity index (CSI) was computed for each of the 36 SDAS items. Table 7 and Supplementary Figure S1 present the ten highest-scoring items. Three items showed clearly elevated sensitivity: SDAS_27 (CSI = 0.803), SDAS_03 (CSI = 0.605), and SDAS_04 (CSI = 0.604). The CSI of SDAS_27 was substantially higher than that of any other item. This item occupies the position immediately after the instructed response item (Item 26), raising the possibility of a position effect in which inattentive responding carries over to adjacent items. Among the mean absolute changes across all 36 items, |Δ*M*| averaged 0.034, |Δ*r*| averaged 0.045, and |Δλ| averaged 0.049.

**Table 7:** Items most sensitive to careless responding.

Item	Factor	|Δ*M*|	|Δ*r*|	|Δλ|	CSI	Item type
SDAS_27	Environment	0.053	0.371	0.379	0.803	Standard
SDAS_03	Economy	0.052	0.259	0.294	0.605	Standard
SDAS_04	Economy	0.047	0.253	0.304	0.604	Standard
SDAS_25	Environment	0.071	0.150	0.152	0.373	Standard
SDAS_R_35	Environment	0.150	0.049	0.084	0.283	Reverse-coded
SDAS_R_31	Environment	0.146	0.023	0.055	0.224	Reverse-coded
SDAS_R_08	Economy	0.110	0.035	0.058	0.203	Reverse-coded
SDAS_R_24	Environment	0.131	0.008	0.030	0.169	Reverse-coded
SDAS_R_01	Economy	0.087	0.022	0.049	0.158	Reverse-coded
SDAS_R_10	Economy	0.083	0.019	0.053	0.155	Reverse-coded

CSI, composite sensitivity index (|ΔM| + |Δr| + |Δλ|); |ΔM|, absolute change in item mean; |Δr|, absolute change in corrected item-total correlation; |Δλ|, absolute change in standardized factor loading. Items with the prefix “SDAS_R_” are reverse-coded. The 10 items with the highest CSI values are shown; all six reverse-coded items in the SDAS appear in this list.

The most striking finding was the concentration of reverse-coded items among the highest CSI values. All six reverse-coded items in the SDAS appeared in the top 10, despite constituting only 16.7% of the item pool (six out of 36). The sensitivity pattern for reverse-coded items differed qualitatively from that of the standard items. For the four standard items in the top 10, the dominant contributors to the CSI were changes in item-total correlations and factor loadings, reflecting weakened relationships with the latent construct. For the six reverse-coded items, by contrast, the primary contributor was the shift in item means, consistent with the expectation that careless responders who fail to process the negation will endorse these items in the same direction as positively worded items, inflating mean scores.

To assess the robustness of these rankings, a standardized version of the index (CSI-*z*) was computed using *z*-scored components. The agreement between the two versions was very high (Spearman's ρ = 0.977, *p* < 0.001; Kendall's τ = 0.901, *p* < 0.001), and the top 10 items were identical across both indices, with no change in their rank order. All six reverse-coded items remained in the top 10 under the CSI-*z* as well. The average rank shift across the 36 items was 1.47 positions, and the largest shift was 8 positions (for SDAS_23, moving from rank 21 to rank 29). These results, illustrated in Supplementary Figure S2, confirm that the disproportionate vulnerability of reverse-coded items and the position effect observed for SDAS_27 are not artifacts of the aggregation method but hold under an alternative weighting scheme.

## Discussion

The present study set out to quantify how careless responding affects the psychometric properties of a widely used attitude measure, the sustainable development awareness scale. By running every analysis in parallel on unscreened and screened samples, we could document the specific ways in which a moderate rate of inattentive responding (11.33%) distorts reliability, factorial validity, measurement invariance, criterion validity, and the detection of group differences. Beyond replicating established patterns of distortion, the study offers two contributions that have received little prior attention: the Composite Sensitivity Index as a tool for identifying the most vulnerable items, and the observation of a position effect tied to the placement of an attention check.

The 11.33% prevalence rate falls squarely within the 8% to 12% range reported across diverse student samples (Curran, 2016; Meade and Craig, 2012; Ward and Meade, 2023), confirming that careless responding is not unique to any single survey format or cultural context. That said, a single instructed response item almost certainly provides a conservative estimate of the overall level of inattention in a dataset. Different detection methods tend to flag overlapping but non-identical sets of respondents (Curran, 2016; Goldammer et al., 2024; Meade and Craig, 2012), and some forms of disengagement, such as satisficing or partial inattention, would go undetected by an item that only tests whether respondents follow a specific instruction. The demographic predictors identified here, namely academic program and student community membership, accounted for only a small share of the variance in careless responding status. This aligns with the broader finding that while individual and situational differences contribute to response quality, careless responding remains difficult to predict from demographic characteristics alone (Bowling et al., 2016; Ward and Meade, 2023). From a practical standpoint, the implication is clear: *post-hoc* data quality screening cannot be replaced by attempts to selectively recruit more attentive respondents.

The reliability findings are consistent with the well-established pattern that careless responses introduce error variance, depressing inter-item correlations and thereby reducing coefficient alpha (Huang et al., 2012; Maniaci and Rogge, 2014). What the present data add to this picture is evidence of differential impact across subscales. The economy and environment subscales, both of which contain reverse-coded items, showed gains in alpha following screening (0.073 and 0.059, respectively), while the society subscale, which has no reverse-coded items, was virtually unaffected. This subscale-level pattern suggests that the reliability costs of careless responding are concentrated in content domains where item wording places additional cognitive demands on the respondent. The gain in hierarchical omega (from 0.781 to 0.809) and the ω*h*/ω*t* ratio of 0.86 in the screened sample further indicate that the general factor underlying the SDAS becomes more clearly defined once careless responses are removed, strengthening the case for computing and interpreting a total score (Reise et al., 2013).

The factorial validity results follow a similar logic. Screening was associated with modest differences in model fit, with CFI, TLI, and SRMR each trending in the direction of better fit and RMSEA essentially unchanged. The mean standardized factor loading across the 36 items was also higher in the screened sample, and the majority of items showed higher loadings after screening. These findings converge with Woods's (2006) simulation work showing that careless responses to reverse-coded items can lead confirmatory factor analyses to reject valid models, and with Kam and Meyer's (2015) demonstration that careless responding can alter the apparent dimensionality of a scale. The improvement in AVE values for the economy and environment factors points to reduced measurement error at the factor level, even though both values remained below the conventional 0.50 cutoff.

The measurement invariance results offer what is perhaps the clearest evidence that careless responding is not simply noise. Metric invariance held across response quality groups, indicating that attentive and careless participants yield similar factor loadings. Scalar invariance, however, was not supported, meaning that the two groups differed systematically in their levels of item endorsement. This pattern is consistent with the interpretation that careless responders bring biases such as acquiescence or midpoint responding that shift observed scores independently of the respondent's true position on the construct (Huang et al., 2012; Woods, 2006). The practical consequence is that mean score comparisons across datasets containing different proportions of careless responders are liable to be biased (Goldammer et al., 2020), a concern that applies both to cross-study comparisons and to longitudinal designs in which screening practices change over time.

The effects of careless responding extended to group comparisons and criterion validity, though the magnitudes were smaller. The overall score distributions changed very little after screening, indicating that removing roughly 11% of the sample did not distort the central tendency of the data. Within the screened sample, however, the number of significant grade-level *post-hoc* contrasts doubled, with new differences emerging between second- and fourth-year and between third- and fourth-year students. This doubling of detectable contrast illustrates how removing careless responders can increase the sensitivity of group comparisons by reducing within-group error variance. Volunteer status and community membership comparisons also showed modest gains in effect size. Taken together, these findings illustrate the power-reducing effect of careless responding described by Maniaci and Rogge (2014): by inflating within-group error variance, careless responses raise the bar for detecting genuine differences. The effect was not uniform, however; department-level comparisons were essentially unchanged, most likely because careless responding was not evenly distributed across programs, as our chi-square analyses confirmed.

Criterion validity correlations followed the expected direction. Both SDAS-PSRS and SDAS-OVC correlations were modestly larger in the screened sample than unscreened sample, though the differences did not reach statistical significance. The small size of these gains likely reflects the relatively low prevalence rate in the present sample; in samples where a larger proportion of respondents are careless, the attenuation of inter-scale correlations would be expected to be more pronounced (Credé, 2010). The adjusted *R*^2^ for the regression of SDAS on PSRS subscales was also slightly larger in the screened sample, providing additional evidence that screening sharpens the relationship between theoretically related constructs, even if the absolute improvements are small. Latent correlations estimated from a five-factor structural model confirmed the same pattern at the measurement-error-corrected level, with small post-screening increases paralleling those observed at the manifest level.

The composite sensitivity index proved to be the most informative analysis for understanding which features of an item make it susceptible to careless responding. The dominant finding was the over-representation of reverse-coded items among the highest CSI values: every one of the six reverse-coded items ranked among the ten highest CSI values, a striking over-representation given that these items constitute only 16.7% of the pool. This result provides empirical support for the theoretical argument that negatively worded items impose additional cognitive demands that careless respondents fail to meet (van Sonderen et al., 2013; Woods, 2006), and it resonates with Steger et al.'s (2023) critique that reverse coding, while intended to reduce acquiescence, can paradoxically introduce measurement error. What the CSI adds to this discussion is a quantitative demonstration that the vulnerability of reverse-coded items is not limited to a single indicator. For these items, the primary driver of the CSI was the shift in item means, consistent with the expectation that careless respondents endorse reverse-coded items as though they were positively worded. For the standard items that appeared in the top 10, by contrast, the CSI was driven primarily by changes in item-total correlations and factor loadings, reflecting a different mechanism of distortion, namely a weakening of the item's relationship with the underlying construct.

A further consideration concerns why particular standard items also showed elevated CSI values. Stosic et al. (2024) have argued that when careless responders tend to select responses toward the positive end of the scale, the magnitude of distortion on any given item depends on how much room there is for the observed mean and item-total correlation to shift. On items with already high attentive-group means, such as SDAS_03 and SDAS_04, acquiescent responding cannot move the mean much further, but it can still weaken the item's relationship with the underlying construct when careless responders endorse the item for reasons unrelated to its content. This is consistent with the pattern we observed: for these standard items, the shift in item-total correlations and factor loadings was substantially larger than the shift in item means. An additional observation is that, in the present scale, items with the lowest attentive-group means were almost exclusively reverse-coded items, which means that the low-mean mechanism described by Stosic et al. (2024) operates here primarily through the reverse-coded subset rather than as a separate effect on standard items. Item-level vulnerability therefore appears to reflect a combination of features, including reverse wording and content-based response patterns, rather than a single mechanism. Disentangling the relative contributions of these factors is an important direction for future work.

The robustness check using the standardized CSI-*z* confirmed that these rankings are not an artifact of the aggregation method. Rank-order agreement between the two versions was very high, and the top 10 items were identical under both approaches. This degree of consistency indicates that the CSI rankings do not depend on the specific metric on which the components are measured, and it supports the use of the unstandardized CSI as a practical heuristic for flagging vulnerable items without requiring elaborate standardization procedures.

The position effect observed for SDAS_27 deserves separate discussion. This item, placed immediately after the instructed response item, showed sensitivity far exceeding that of any other item in the scale. One plausible explanation is a position effect, in which respondents who failed the attention check continued to respond with comparable or even greater inattention on the next item. This interpretation would be consistent with the view that careless responding reflects a sustained state rather than a momentary lapse (Ward and Meade, 2023), and Gummer et al.'s (2021) finding that exposure to an instructed response item does not alter response behavior among attentive participants. At the same time, a position-effect account is necessarily tentative in the present design. The position of the instructed response item was fixed, which means that the elevated CSI for SDAS_27 could in principle reflect features of the item itself rather than its placement. A stronger test would require varying the position of the attention check across forms and examining whether items adjacent to it consistently show elevated sensitivity regardless of which content items happen to occupy those positions. Until such evidence is available, the position effect observed here should be treated as a hypothesis worth investigating rather than as a settled finding. A practical implication that nonetheless follows from either interpretation is that items adjacent to attention checks warrant particular scrutiny, and that where possible, critical items should not be placed immediately after attention checks until the underlying mechanism is better understood.

The findings carry several broader implications. For scale developers, the concentration of careless responding effects in reverse-coded items raises the question of whether the benefits of reverse coding (reduced acquiescence, response consistency checks) outweigh its costs (inflated error, weakened factor structure) in contexts where routine screening for careless responding is not standard practice. For applied researchers, the doubling of significant *post-hoc* contrasts after screening illustrates that data cleaning is not merely a methodological formality but a step that can meaningfully affect substantive conclusions. For researchers interested in cross-cultural measurement, the replication of established prevalence rates and psychometric distortion patterns in a Turkish university sample suggests that the basic phenomena of careless responding are not confined to Western settings. Grau et al. (2019), in one of the few direct cross-country comparisons of careless responding, reported meaningful variation across national samples in both prevalence and the performance of different detection indices, and the present findings add one further data point to that developing literature. Future research should continue to investigate whether cultural factors moderate the specific mechanisms involved.

### Limitations and future directions

Several limitations should be considered when interpreting these findings. The most consequential is that careless responding was identified using a single instructed response item. While this method provides an unambiguous criterion for classification, it captures only one form of inattention and has two specific weaknesses that merit emphasis. First, an item that asks respondents to select a specific non-midpoint category will not flag respondents who straightline through the middle of the scale; such midpoint respondents appear to be a non-trivial subgroup in survey data (Pokropek, 2026), and by design they pass the kind of attention check used here. Second, relying on a single attention check substantially increases the probability that careless respondents will pass it by chance; (Muszyński 2023) has argued that multiple attention checks distributed across the instrument provide meaningfully better classification than any single item can. More broadly, the instructed response item may miss respondents who satisfice or who disengage in ways that do not involve overt failure to follow instructions (Krosnick, 1991), and there is also a risk that some respondents who failed the item did so for reasons other than carelessness, such as confusion about the instruction, deliberate noncompliance, or misinterpretation of item wording (Curran and Hauser, 2019). Silber et al. (2022) found that roughly 61% of respondents who failed an instructed response item reported having noticed it, suggesting that a non-trivial share of failures reflect something other than pure inattention. In the present data, we were able to partially offset some of these concerns by showing that the instructed-item classification converged with *post-hoc* indicators (longstring and even-odd consistency). Nonetheless, future studies would benefit from employing multiple screening methods in combination, including both *a priori* indicators (instructed response items, bogus items) and *post hoc* indices (Mahalanobis distance, longstring analysis, psychometric consistency), to obtain a more comprehensive picture of data quality (Curran, 2016; DeSimone et al., 2015; Goldammer et al., 2024).

Because the present study used a single instructed response item as the primary classification criterion, a supplementary robustness analysis was conducted in which the careless definition was broadened to include longstring and even-odd consistency indicators alongside the instructed response item (see Results and Supplementary Table S5). The extended classification identified 29 additional cases beyond the 126 flagged by the instructed response item, and the substantive conclusions of the study were largely preserved under this alternative definition: reliability changes remained concentrated in subscales containing reverse-coded items, confirmatory factor analysis indices moved in the same direction, and the measurement invariance pattern was identical, with metric but not scalar invariance supported. Two outcomes diverged. The correlation between SDAS and PSRS and the variance explained by PSRS subscales in the regression changed less under the extended criterion, and in the case of *R*^2^ the small gain observed under the instructed-item classification did not emerge. These divergences are consistent with the view that different careless responding indices capture partially distinct response phenomena: the instructed response item detects noncompliance with explicit instructions, the longstring indicator detects extended runs of identical responses, and the even-odd index detects within-person inconsistency. The 29 additional cases flagged under the extended criterion were predominantly straightliners or inconsistent responders who nonetheless complied with the instructed response item, and their exclusion appears to reduce variance in the criterion measures as well as in the SDAS, dampening the observed criterion correlations. The broader implication is that the instructed response item, despite its minimalism, identifies the subgroup whose responses most strongly affect the psychometric properties examined here. This does not remove the need for multi-indicator approaches in future research; indeed, such approaches remain the methodological ideal. It does, however, suggest that the present findings are not an artifact of having used a single indicator.

The sample was drawn from students at a single public university in Türkiye, which limits the generalizability of the findings. The gender composition of roughly four-fifths women reflects the demographics of the teacher education programs from which participants were recruited but may not be representative of other student populations or of non-student samples. Cross-cultural research has suggested that attention check performance and response styles can vary across national and cultural contexts (Kay and Saucier, 2023; Ozaki, 2024), and replication in more diverse samples is needed.

The study relied on paper-and-pencil administration, which differs from the online survey context in which much of the careless responding literature has been produced. Paper-and-pencil surveys are administered in a controlled, proctored setting, which may reduce certain forms of careless responding (such as speeding or multitasking) while leaving others intact. Whether the patterns observed here hold for computer-administered surveys, where response time data can be captured and used as an additional screening indicator, remains an open question (Gummer et al., 2021).

The CSI, while useful as a heuristic, is a novel index whose properties have not yet been evaluated across different instruments and samples. The robustness check using CSI-*z* provides initial evidence of rank stability, but future research should examine whether CSI rankings replicate when the index is applied to scales with different item formats, response options, or proportions of reverse-coded items. Investigation of whether items with high CSI values can be revised to reduce their vulnerability to careless responding would also be a valuable line of work.

Finally, the growing interest in model-based and machine learning approaches to careless responding detection suggests that more sophisticated classification methods may prove useful for identifying patterns that traditional indices miss. Model-based approaches that exploit response time information, such as the mixture item response model of Ulitzsch et al. (2022), provide a particularly promising framework because they can distinguish careless from effortful responses without relying on the accuracy of any single attention check item. Supervised machine learning techniques offer a complementary direction by training classifiers on known careless response patterns (Pokropek, 2026; Schroeders et al., 2022). At present, however, the generalizability of these methods across heterogeneous survey contexts remains limited, and further development is needed before they can be recommended as routine tools.

## Conclusion

This study demonstrates that a careless responding rate of 11.33%, well within the range commonly reported in student samples, is sufficient to attenuate reliability, degrade factorial validity, prevent the establishment of scalar measurement invariance, weaken criterion-related validity correlations, and reduce the sensitivity of group comparisons. These effects are not random; they are concentrated in reverse-coded items and in items placed adjacent to attention checks, revealing identifiable vulnerabilities that researchers and scale developers can address through thoughtful survey design. The composite sensitivity index provides a practical means of identifying items most affected by careless responding, and the close agreement between the CSI and its standardized counterpart (CSI-*z*) supports its use as a screening heuristic. The broader implication is that data quality screening should be treated not as an optional procedural step but as a necessary component of any research program that relies on self-report measures.

## Data Availability

The datasets presented in this study can be found in online repositories. The names of the repository/repositories and accession number(s) can be found at: https://osf.io/d5hb4?view_only=7812369a65be424f83824e0dc79aa805.
