# Supplementary material for: More than just noise: careless responding and its systematic effects on reliability, validity, and measurement invariance
Source: Front Psychol. 2026 May 15;17:1815225. doi: 10.3389/fpsyg.2026.1815225 (PMC13219303; doi:10.3389/fpsyg.2026.1815225)
Supplement: Supplementary file 1 [file Data_Sheet_1.docx]

Supplementary Material

For

More Than Just Noise: Careless Responding and Its Systematic Effects on Reliability, Validity, and Measurement Invariance

# Supplementary Tables

**Table S1**

*Binary Logistic Regression Predicting Careless Responding*

| **Predictor** | **B** | **SE** | **OR** | **95% CI** | **p** |
| --- | --- | --- | --- | --- | --- |
| (Intercept) | −3.76 | 0.70 | 0.02 | [0.01, 0.08] | <.001 |
| **Gender (ref: Female)** |  |  |  |  |  |
| Male | 0.28 | 0.23 | 1.32 | [0.83, 2.07] | .230 |
| **Grade (ref: 1st Year)** |  |  |  |  |  |
| 2nd Year | 0.43 | 0.28 | 1.54 | [0.89, 2.69] | .127 |
| 3rd Year | −0.06 | 0.31 | 0.94 | [0.51, 1.72] | .843 |
| 4th Year | −0.12 | 0.30 | 0.89 | [0.49, 1.61] | .699 |
| **Department (ref: SciED)** |  |  |  |  |  |
| SSE | 1.32 | 0.69 | 3.76 | [1.07, 17.44] | .054 |
| GPC | 1.19 | 0.63 | 3.29 | [1.10, 14.20] | .059 |
| PE | 1.89 | 0.65 | 6.62 | [2.13, 29.20] | .003 |
| SpE | 2.10 | 0.63 | 8.14 | [2.73, 35.12] | <.001 |
| ELT | 1.46 | 0.64 | 4.31 | [1.40, 18.87] | .023 |
| TLT | 0.42 | 0.85 | 1.52 | [0.27, 8.61] | .623 |
| EMT | 1.22 | 0.68 | 3.38 | [0.99, 15.53] | .073 |
| CT | 1.23 | 0.67 | 3.42 | [1.01, 15.63] | .069 |
| **Volunteer (ref: Yes)** |  |  |  |  |  |
| No | −0.17 | 0.26 | 0.85 | [0.51, 1.44] | .523 |
| **Community (ref: Yes)** |  |  |  |  |  |
| No | 0.49 | 0.20 | 1.64 | [1.10, 2.45] | .016 |

*Note.* N = 1,112. The dependent variable is careless responding status (1 = careless, 0 = attentive). Reference categories for categorical predictors are: Female (gender), 1st year (grade), Science Education / SciED (department), Yes (volunteer), and Yes (community membership). B = unstandardized logistic regression coefficient; SE = standard error; OR = odds ratio; 95% CI = 95% confidence interval for the odds ratio. Department abbreviations: **SciED** = Science Education; **SSE** = Social Studies Education; **GPC** = Guidance and Psychological Counseling; **PE** = Preschool Education; **SpE** = Special Education; **ELT** = English Language Teaching; **TLT** = Turkish Language Teaching; **EMT** = Elementary Mathematics Teaching; **CT** = Classroom Teaching. Model fit: χ²(14) = 35.58, p = .001; McFadden's pseudo-R² = .045.

**Table S2**

*Standardized Factor Loadings by Sample for All SDAS Items*

| **Factor** | **Item** | **λ Uns** | **λ Scr** | **Δλ** |
| --- | --- | --- | --- | --- |
| Economy | SDAS_R_01 | 0.13 | 0.179 | 0.049 |
|  | SDAS_02 | 0.461 | 0.465 | 0.004 |
|  | SDAS_03 | 0.273 | 0.567 | 0.294 |
|  | SDAS_04 | 0.352 | 0.656 | 0.304 |
|  | SDAS_05 | 0.676 | 0.69 | 0.014 |
|  | SDAS_06 | 0.754 | 0.747 | −.007 |
|  | SDAS_07 | 0.544 | 0.527 | −.017 |
|  | SDAS_R_08 | 0.221 | 0.279 | 0.058 |
|  | SDAS_09 | 0.596 | 0.57 | −.026 |
|  | SDAS_R_10 | 0.091 | 0.144 | 0.053 |
|  | SDAS_11 | 0.484 | 0.463 | −.021 |
|  | SDAS_12 | 0.536 | 0.515 | −.021 |
|  | SDAS_13 | 0.704 | 0.694 | −.010 |
| Society | SDAS_14 | 0.551 | 0.569 | 0.018 |
|  | SDAS_15 | 0.781 | 0.786 | 0.005 |
|  | SDAS_16 | 0.758 | 0.747 | −.011 |
|  | SDAS_17 | 0.716 | 0.712 | −.004 |
|  | SDAS_18 | 0.732 | 0.733 | 0.001 |
|  | SDAS_19 | 0.331 | 0.309 | −.022 |
|  | SDAS_20 | 0.563 | 0.557 | −.006 |
|  | SDAS_21 | 0.758 | 0.763 | 0.005 |
|  | SDAS_22 | 0.716 | 0.718 | 0.002 |
| Environment | SDAS_23 | 0.544 | 0.562 | 0.018 |
|  | SDAS_R_24 | 0.083 | 0.113 | 0.03 |
|  | SDAS_25 | 0.3 | 0.452 | 0.152 |
|  | SDAS_27 | 0.391 | 0.77 | 0.379 |
|  | SDAS_28 | 0.75 | 0.784 | 0.034 |
|  | SDAS_29 | 0.776 | 0.782 | 0.006 |
|  | SDAS_30 | 0.704 | 0.72 | 0.016 |
|  | SDAS_R_31 | 0.32 | 0.375 | 0.055 |
|  | SDAS_32 | 0.655 | 0.656 | 0.001 |
|  | SDAS_33 | 0.753 | 0.756 | 0.003 |
|  | SDAS_34 | 0.763 | 0.773 | 0.01 |
|  | SDAS_R_35 | 0.407 | 0.491 | 0.084 |
|  | SDAS_36 | 0.567 | 0.553 | −.014 |
|  | SDAS_37 | 0.456 | 0.466 | 0.01 |

*Note.* Uns = unscreened (N = 1,112); Scr = screened (n = 986); λ = standardized factor loading; Δλ = λ(Scr) − λ(Uns). Items with the prefix “SDAS_R_” are reverse-coded. Loadings are estimated from separate confirmatory factor analyses fitted to each sample using robust maximum likelihood. SDAS_26 is not included as it is the instructed response item used for careless responding detection.

**Table S3**

*Effect Sizes for Group Comparisons by Sample*

| Independent Samples T-Tests (Cohen’s d with 95% CI) | | | | | | | |
| --- | --- | --- | --- | --- | --- | --- | --- |
|  | Unscreened | | | Screened | | | Δd |
| Comparison | d | 95% CI | p | d | 95% CI | p |  |
| Gender (Female vs Male) | | | | | | | |
| SDAS Total | 0.289 | [0.14, 0.44] | <.001 | 0.275 | [0.12, 0.43] | .001 | −0.014 |
| Volunteer Status (Yes vs No) | | | | | | | |
| SDAS Total | 0.205 | [0.04, 0.37] | .006 | 0.242 | [0.07, 0.42] | .002 | 0.037 |
| Community Member (Yes vs No) | | | | | | | |
| SDAS Total | 0.128 | [0.01, 0.25] | .033 | 0.185 | [0.06, 0.31] | .004 | 0.057 |
| One-Way ANOVAs (η² and ω²) | | | | | | | |
|  | Unscreened | | | Screened | | | Δη² |
| Grouping Variable | η² | ω² | p | η² | ω² | p |  |
| Grade Level (4 groups) | .011 | .009 | .006 | .016 | .013 | .001 | .005 |
| F statistic | F(3, 1108) = 4.19 | |  | F(3, 982) = 5.35 | | |  |
| Significant post-hoc pairs | 2 |  |  | 4 |  |  | +2 |
| Department (9 groups) | .021 | .014 | .003 | .020 | .012 | .011 | −.001 |
| F statistic | F(8, 1103) = 2.99 | | | F(8, 977) = 2.50 | | | |
| Significant post-hoc pairs | 2 |  |  | 2 |  |  | 0 |

*Note.* d = Cohen’s d; η² = eta-squared; ω² = omega-squared; Δ = change (Screened − Unscreened). Screened vs Unscreened Means compares the two samples directly; all other comparisons are within-sample group differences. Post-hoc tests used Games-Howell correction.

**Table S4.**

*Latent and Manifest Correlations Between SDAS Subscales and Criterion Measures by Sample*

| **Pair** | **Manifest r (Uns)** | **Latent r (Uns)** | **Manifest r (Scr)** | **Latent r (Scr)** | **Δ Manifest** | **Δ Latent** |
| --- | --- | --- | --- | --- | --- | --- |
| Economy – PSRS | 0.260 | 0.365 | 0.302 | 0.376 | 0.042 | 0.011 |
| Society – PSRS | 0.305 | 0.374 | 0.307 | 0.379 | 0.002 | 0.005 |
| Environment – PSRS | 0.347 | 0.444 | 0.380 | 0.454 | 0.033 | 0.010 |
| Economy – OVC | 0.211 | 0.258 | 0.247 | 0.283 | 0.036 | 0.025 |
| Society – OVC | 0.247 | 0.265 | 0.265 | 0.285 | 0.018 | 0.02 |
| Environment – OVC | 0.288 | 0.317 | 0.331 | 0.339 | 0.043 | 0.022 |
| PSRS – OVC | 0.541 | 0.575 | 0.534 | 0.573 | −.007 | −.002 |

*Note.* Uns = unscreened (N = 1,112); Scr = screened (n = 986). Manifest correlations are zero-order Pearson correlations between scale total scores. Latent correlations are estimated from a five-factor structural equation model in which Economy, Society, Environment, PSRS, and OVC are specified as separate latent factors with freely estimated covariances. The five-factor model was fitted separately to each sample using robust maximum likelihood. PSRS = Personal Social Responsibility Scale; OVC = Obligation to Volunteer as Commitment scale. All correlations are significant at p < .001.

**Table S5.**

*Robustness of Key Psychometric Outcomes to Careless Responding Definition*

| **Outcome** | **Unscreened** | **IRI-Screened** | **Extended-Screened** | **Δ IRI** | **Δ Extended** |
| --- | --- | --- | --- | --- | --- |
| *Sample size (n)* | 1,112 | 986 | 957 | — | — |
| *Careless (n)* | — | 126 (11.3%) | 155 (13.9%) | — | — |
| **Cronbach's α** | | | | | |
| SDAS Total | 0.925 | 0.938 | 0.935 | 0.013 | 0.011 |
| SDAS Economy | 0.766 | 0.812 | 0.807 | 0.046 | 0.041 |
| SDAS Society | 0.872 | 0.871 | 0.877 | −.001 | 0.004 |
| SDAS Environment | 0.848 | 0.881 | 0.873 | 0.034 | 0.026 |
| **CFA Fit Indices** | | | | | |
| CFI | 0.897 | 0.91 | 0.903 | 0.013 | 0.006 |
| TLI | 0.891 | 0.904 | 0.897 | 0.013 | 0.006 |
| RMSEA | 0.046 | 0.047 | 0.047 | 0 | 0.001 |
| SRMR | 0.043 | 0.041 | 0.042 | −.002 | −.001 |
| **Criterion Correlations** | | | | | |
| *r* (SDAS–PSRS) | 0.352 | 0.376 | 0.349 | 0.025 | −.003 |
| *r* (SDAS–OVC) | 0.288 | 0.321 | 0.305 | 0.033 | 0.017 |
| **Measurement Invariance (CFI)** | | | | | |
| Configural | — | 0.878 | 0.876 | — | — |
| Metric (ΔCFI) | — | −.002 | −.004 | — | — |
| Scalar (ΔCFI) | — | −.020 | −.021 | — | — |
| Metric supported | — | Yes | Yes | — | — |
| Scalar supported | — | No | No | — | — |
| **Regression (PSRS → SDAS Total)** | | | | | |
| R² | 0.182 | 0.188 | 0.162 | 0.007 | −.020 |
| Adjusted R² | 0.178 | 0.184 | 0.157 | 0.006 | −.021 |

*Note.* This table compares the psychometric results obtained under two definitions of careless responding. The IRI-Screened column reports results after excluding the 126 respondents (11.3%) flagged by the instructed response item alone (the primary classification used throughout the main manuscript). The Extended-Screened column reports results after excluding the 155 respondents (13.9%) flagged by a combined criterion of the instructed response item, the longstring index (cutoff ≥ 20.72, two standard deviations above the sample mean), and the even-odd consistency index (cutoff ≥ 1.707, two standard deviations above the sample mean), combined with a logical OR. Cutoffs for the two post-hoc indices were computed on the unscreened sample. Δ IRI = IRI-Screened value minus Unscreened value. Δ Extended = Extended-Screened value minus Unscreened value. For measurement invariance, ΔCFI values are calculated relative to the configural model, and the criterion ΔCFI ≥ −.010 (Cheung & Rensvold, 2002) was used to evaluate invariance support. The extended criterion flagged 29 additional respondents beyond those flagged by the instructed response item alone. Of the 29 additional cases, 12 were flagged by longstring only, 17 by even-odd only, and no respondents were flagged by both longstring and even-odd without also failing the instructed response item. An intersection (AND) combination of the three indicators would have flagged only 7 respondents, a sample size inadequate for the planned subgroup analyses. SDAS = Sustainable Development Awareness Scale; PSRS = Personal Social Responsibility Scale; OVC = Obligation to Volunteer as Commitment scale; CFI = comparative fit index; TLI = Tucker–Lewis index; RMSEA = root mean square error of approximation; SRMR = standardized root mean square residual.


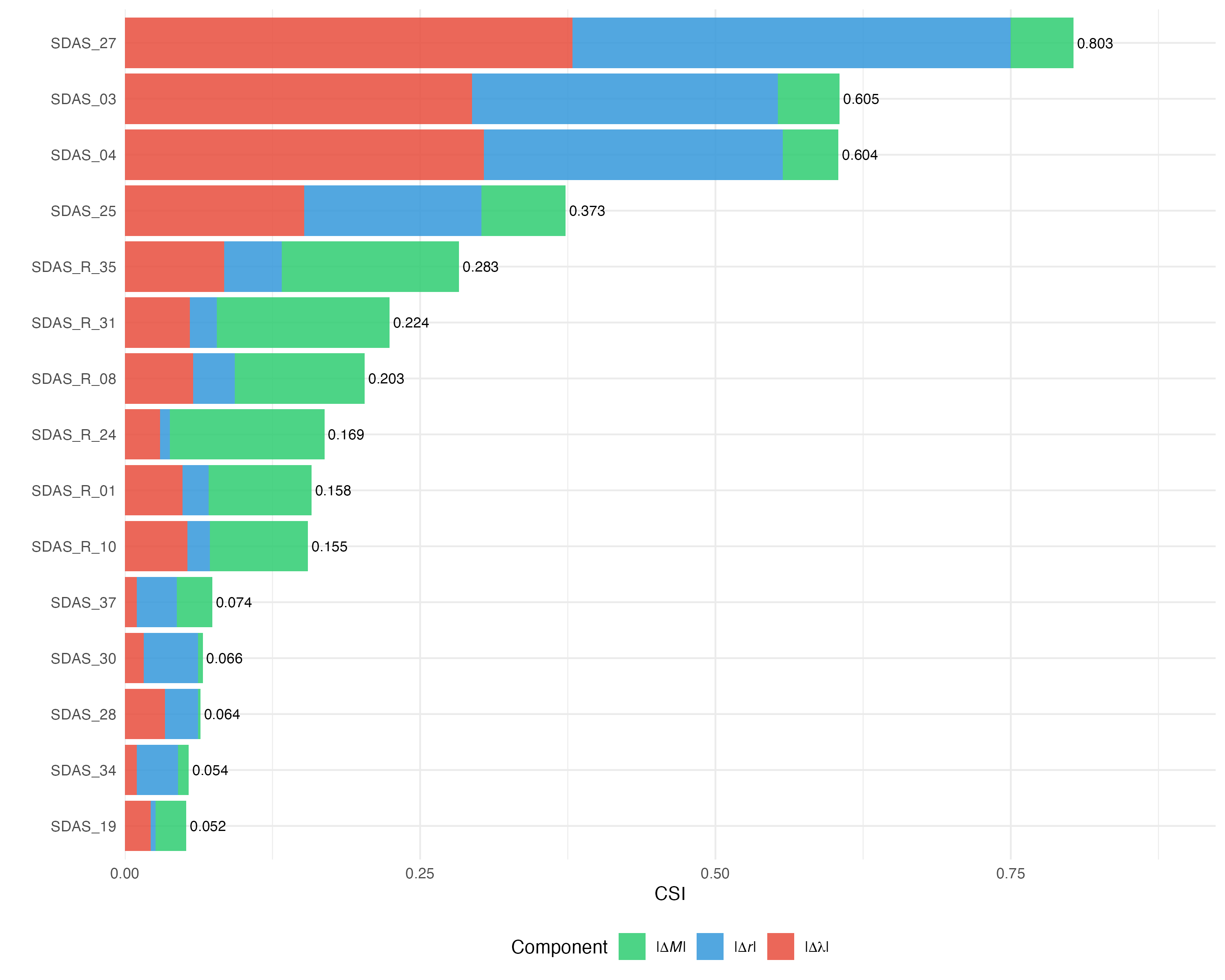


**Supplementary Figure S1.** Composite Sensitivity Index (CSI) scores for the 15 SDAS items with the highest values, shown as stacked bars. Each bar is decomposed into three components: the absolute change in item mean (|ΔM|), the absolute change in corrected item-total correlation (|Δr|), and the absolute change in standardized factor loading (|Δλ|). Items with the prefix "SDAS_R_" are reverse-coded. All six reverse-coded items in the SDAS appear within the top 10 CSI values. SDAS_27, which occupies the position immediately after the instructed response item, shows the highest CSI value, driven primarily by changes in item-total correlations and factor loadings rather than by changes in item means. The reverse-coded items, by contrast, show CSI values driven primarily by changes in item means.


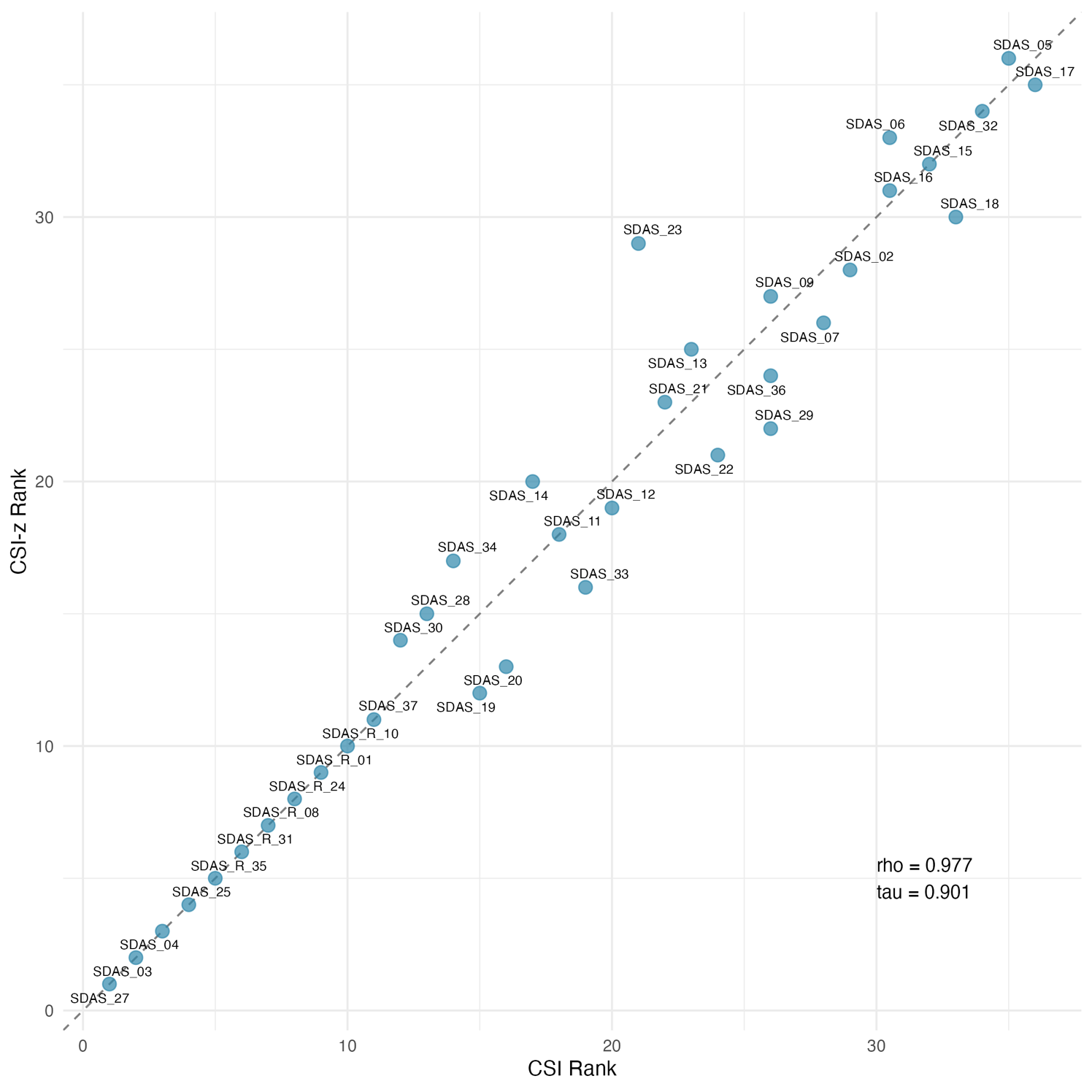


**Supplementary Figure S2*.*** Comparison of CSI and CSI-z rankings across all 36 SDAS items. Each point represents one item, with its rank under the unstandardized Composite Sensitivity Index (CSI) on the horizontal axis and its rank under the standardized version (CSI-z) on the vertical axis. The diagonal line indicates perfect rank agreement. Spearman's ρ = .977, Kendall's τ = .901. The 10 items with the highest CSI values were identical under both versions and appeared in the same rank order, supporting the robustness of the CSI rankings to the choice of aggregation metric.
